# Physical Activity in Deprived Communities in London: Examining Individual and Neighbourhood-Level Factors

**DOI:** 10.1371/journal.pone.0069472

**Published:** 2013-07-26

**Authors:** Paul Watts, Gemma Phillips, Mark Petticrew, Richard Hayes, Christian Bottomley, Ge Yu, Elena Schmidt, Patrick Tobi, Derek Moore, Caroline Frostick, Karen Lock, Adrian Renton

**Affiliations:** 1 Institute for Health and Human Development, University of East London, London, United Kingdom; 2 Department of Health Services Research and Policy, London School of Hygiene and Tropical Medicine, London, United Kingdom; 3 Department of Social and Environmental Health Research, London School of Hygiene and Tropical Medicine, London, United Kingdom; 4 Tropical Epidemiology Group, Department of Infectious Disease Epidemiology, Faculty of Epidemiology and Population Health, London School of Hygiene and Tropical Medicine, London, United Kingdom; 5 Leeds Institute of Health Sciences, University of Leeds, Leeds, Yorkshire, United Kingdom; 6 Sightsavers, Haywards Heath, West Sussex, United Kingdom; 7 Institute for Research on Child Development, University of East London, Water Lane, London, United Kingdom; Chancellor College, University of Malawi, Malawi

## Abstract

**Introduction:**

The objectives of this study were to examine relationships between neighbourhood-level and individual-level characteristics and physical activity in deprived London neighbourhoods.

**Methods:**

In 40 of the most deprived neighbourhoods in London (ranked in top 11% in London by Index of Multiple Deprivation) a cross-sectional survey (n = 4107 adults aged > = 16 years), neighbourhood audit tool, GIS measures and routine data measured neighbourhood and individual-level characteristics.

The binary outcome was meeting the minimum recommended (CMO, UK) 5×30 mins moderate physical activity per week. Multilevel modelling was used to examine associations between physical activity and individual and neighbourhood-level characteristics.

**Results:**

Respondents living more than 300 m away from accessible greenspace had lower odds of achieving recommended physical activity levels than those who lived within 300 m; from 301–600 m (OR = 0.7; 95% CI 0.5–0.9) and from 601–900 m (OR = 0.6; 95% CI 0.4–0.8). There was substantial residual between-neighbourhood variance in physical activity (median odds ratio = 1.7). Other objectively measured neighbourhood-level characteristics were not associated with physical activity levels.

**Conclusions:**

Distance to nearest greenspace is associated with meeting recommended physical activity levels in deprived London neighbourhoods. Despite residual variance in physical activity levels between neighbourhoods, we found little evidence for the influence of other measured neighbourhood-level characteristics.

## Introduction

Regular physical activity is effective in reducing the risk of premature death and in preventing the development of many chronic diseases [1]. It is currently recommended by the Chief Medical Officer for England that adults take some moderate or vigorous physical activity every day and a minimum of 150 minutes per week [2]. However, these minimum recommended levels of physical activity are met by only 39% of men and 29% of women in England [3].

The majority of research on factors associated with physical activity has focused on individual-level factors [4] and most interventions have aimed to increase participation in physical activity through the modification of one or more of these individual-level factors [5]. However, social-ecological models propose that physical activity levels are determined not exclusively by individual-level factors but through the interaction of individual-level factors and characteristics of the places in which people live [6], [7]. Indeed, there is a growing evidence base to suggest that neighbourhoods may support or constrain opportunities to lead physically active lifestyles through the influence of a range of neighbourhood-level factors. Collectively, these factors are often referred to in the literature as the ‘physical’ or ‘built’ environment, but may also include elements of the social environment. Several comprehensive reviews of this evidence base have summarised the key relationships between neighbourhood-level variables and physical activity levels [8]–[12].

The vast majority of this research has been published outside the UK (predominantly in the United States and Australia) and there is evidence to suggest that the findings of these studies may not be generalizable between countries [13]. This study uses a social-ecological approach [6], [7] to investigate the influence of individual and neighbourhood-level factors on individual-level physical activity levels in deprived neighbourhoods in London (UK). Our approach incorporates many of the common recommendations for further research that have been identified in reviews of the evidence base, which have recently been summarised [14]. The recommendations that we seek to address are: (1) incorporation of both objective and perceived measures of neighbourhood-level factors; (2) examination of population sub-groups (e.g. low socioeconomic status); (3) application of multi-level models in data analysis; and (4) use of audits to increase specificity in the measurement of physical neighbourhood-level factors. Through the application of these recommendations, this study seeks to address the following research questions:

Research question 1: Are individual and neighbourhood-level characteristics of deprived neighbourhoods in London associated with individual-level physical activity?

Research Question 2: How much variation in physical activity levels is there between neighbourhoods?

## Materials and Methods

### Overview of the study

This study utilises an observational design based on a cross-sectional survey. The survey was conducted at baseline within the Well London programme cluster randomised controlled trial, which includes twenty pairs of control and intervention neighbourhoods across twenty boroughs [15]. The survey was conducted before delivery of the interventions and data were collected on the health and wellbeing of adults (16+ years) and on the physical, structural and social aspects of the neighbourhoods in which they lived. Additional routine data at the neighbourhood-level were collated to complement data on the physical and structural aspects of the neighbourhoods. Data collection and analysis procedures are described in more detail below.

### Neighbourhood definition and selection

The setting for this study was 40 of the most deprived neighbourhoods in London (2 in each of 20 London boroughs). Neighbourhoods were defined as census lower super-output areas (LSOA) selected for inclusion in the Well London trial because they are ranked within the top 11% for deprivation in London as measured by the English indices of deprivation [16]. LSOAs are commonly used administrative areas with a minimum of 1,000 residents and a mean of approximately 1,500 residents.

### Adult survey

Interviewer-administered surveys were conducted in 2008 by trained fieldworkers in households selected at random from the Post Office Address Files for each of the 40 neighbourhoods. At addresses that responded to visits from fieldworkers, every eligible adult (aged 16 years and over) providing written, informed consent was interviewed separately from other participants. Households were classified as non-responding only after fieldworkers had attempted visits on at least five separate days, at varying times of the day.

### Physical activity outcome measure

The household survey included the short form of the International Physical Activity Questionnaire (IPAQ-SF) [17] to produce three categories of physical activity levels: ‘high’, ‘moderate’ and ‘low’. The definition of low physical activity is analogous to not meeting the minimum recommended levels of physical activity [2]. The binary outcome variable for this study was either meeting recommended levels (‘moderate’ or ‘high’ physical activity) or not meeting the levels (‘low’ physical activity).

### Individual-level sociodemographic factors

The household survey collected self-reported measures of sociodemographic characteristics, positive mental wellbeing (The Hope Scale [18]), self-rated health (mobility problems, problems performing usual activities, pain/discomfort, depression/anxiety) from the Euroqol EQ-5D [19]–[21] and social capital (Questions from the Office for National Statistics Social Capital Harmonised Question Set [22], [23]). Results relating to the data collected on social capital have been published previously with an alternative modelling strategy [24] but are presented here for completeness in the context of the wider neighbourhood analysis.

### Resident-reported neighbourhood characteristics

Participants were asked to rate their overall satisfaction with their neighbourhood and the quality of certain aspects of their neighbourhood and asked to rate how safe they feel alone on the streets in their neighbourhood both during the day and at night.

### Neighbourhood audit

Physical and structural neighbourhood-level factors were measured using a systematic social observation tool designed for this study following a review of previously validated tools [25]–[27] and the theoretical literature. This tool is available from the authors on request. Trained observers visited each of the 40 neighbourhoods using a systematic address-based sampling approach to complete a pro-forma in pre-defined segments of the neighbourhoods and a sample of these segments were cross-checked using Google Earth. See Table 1 for details of the items recorded using the audit tool and the scales used.

**Table 1:** Details of the Items and Scales Used From the Neighbourhood Audit Tool.

Neighbourhood Audit Item/Scale	Items included
*Parks and Greenspaces*	Number of communal green spaces and large parks
*Cyclability*	Presence of continuous and non-continuous cycle lanes, speed limit and availability of cycle storage facilities
*Pedestrian infrastructure*	Condition and width of pavements, number of road crossing aids and overpasses/underpasses
*Traffic speed/volume*	Speed limit, number of traffic calming measures, size of roads (number of lanes of traffic)
*Signs of social disorder and incivilities*	Presence and amount of: litter and broken glass; graffiti; vandalised facilities; broken windows; security measures; unattended dogs; large items dumped in public areas; dog foul; needles/syringes/condoms; empty alcohol bottles/cans; signs of home personalisation; greenery; neighbourhood watch signs

### Routine data

Data from the Generalised Land Use Database was used to create a measure of the proportion of each area classified as greenspace, residential, commercial, transport and ‘other’ land uses in each neighbourhood. A land-use-mix index, which provides an indicator of how well land use types such as residential, commercial and greenspace are balanced was calculated using the Herfindahl-Hirschman method described by Smith and Davey [28]. We used a crime indicator from the English Indices of Deprivation 2007 [29] to look at neighbourhood-level crime rates. This indicator includes recorded rates of four categories of crime: burglaries, thefts, violence and criminal damage. A street connectivity index was created from counts of the three-way and four-way junctions within the neighbourhoods, adjusted for the size of the neighbourhoods [28]

### Distance to nearest greenspace

The postcodes of residents who had participated in the household survey were geocoded using Arc GIS Version 9.1 (Environmental Systems Research Institute, Redlands, CA, USA). Publicly accessible and useable greenspaces in close proximity to each of the neighbourhoods were identified visually using aerial images from Google Earth and then the access points to the greenspaces were geocoded using Arc GIS. Only greenspaces larger than 2 hectares were geocoded as areas smaller than this were considered to be of inadequate size for adults to use to be physically active [30]. Judgements as to whether identified greenspaces were accessible (open to the public with at least one access point from a public road or path) and usable (containing walkable paths and/or open, walkable surfaces) were made using methods described by Natural England [31] and Taylor et al [32]. The shortest walking distance from each postcode to the nearest greenspace access point was calculated initially using Ordnance Survey Centre Alignment of Roads (OSCAR) data. All walking routes were examined using Google Earth and Google Street view and subsequently modified if necessary to ensure that the shortest unobstructed walking route was accurately recorded. Walking distance to greenspace was examined by using a categorical variable for walking distance to green space (≤300 m, 301–600 m, 601–900 m, 901–1200 m).

### Access to sport/leisure facilities, food stores and town centres

The English Indices of Deprivation core accessibility measures were used as indicators of the neighbourhood average walking distances to the nearest available food store and town centre [33]. Sport England's Active Places Power Strategic Planning Tool [34] was used to identify the number of sports and leisure facilities within ten minutes walking distance from the centre of the neighbourhoods. All these measures use OSCAR data to calculate the quickest walking route.

### Missing household survey data

Missing data were accounted for using multiple imputation models. The user-written ice commands [35]–[41] in Stata v11.2 (Stata Corp, TX, USA) were used to complete five imputations with ten cycles in each imputation. For the binary physical activity outcome, each item on the short form of the International Physical Activity Questionnaire (IPAQ - SF) was imputed and the overall physical activity score was calculated from these items (See Text S1. for details of how the imputation models were specified). The results presented are from the imputed dataset, but complete case analyses were also conducted (see Table S1 and Table S2).

### Data analysis

Analyses were conducted using Stata v11.2 (Stata Corp, TX, USA). For the individual-level factors, logistic regression models were used to adjust associations between the household survey items (sociodemographic factors, mental wellbeing and resident-reported environmental factors) and physical activity levels for age, sex, ethnicity and LSOA. Logistic regression analyses were also conducted for level-one variables including LSOA as a random effect, but the fixed effects approach was preferred in order to account for potential endogeneity in level-one variables [42]. There were no substantial differences in the point estimates and standard errors between the two analytical approaches so we have presented the fixed effects approach for level-one variables. For the neighbourhood-level factors, logistic regression was also used to adjust for age, sex and ethnicity and LSOA was included in the models as a random effect.

### Median odds ratio

Larsen and colleagues [43], [44] have proposed using the ‘median odds ratio’ to quantify variation between neighbourhoods when examining a binary outcome. The median odds ratio compares pairs of individuals with the same covariates from two randomly selected neighbourhoods. It is the median of the odds ratios comparing the individual with higher propensity for physical activity and the person of lower propensity, and can be calculated using equation 1 below [43], [45], [46].
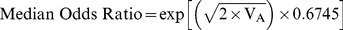
(1)


### Ethical Approval

Ethical approval for all procedures was granted by the University of East London ethics committee in line with the declaration of Helsinki. Written, informed consent was granted by all participants. For participants aged 16 or 17, written, informed consent from both the participant and a parent or guardian was obtained prior to participation (these consent forms were signed by both participants and parents/guardians).

## Results

### Household survey response rates and missing data

The household survey was completed by 4,107 adults. Further information about the study sample is presented elsewhere [15]. The mean response rate at the household-level was 73.3% across the 40 neighbourhoods (standard deviation: 13.9; range: 40.5%–99%) and within responding households the individual-level response rate was 61%.

### Physical activity levels and relationship with individual-level sociodemographic factors

Data from the IPAQ-SF indicated that 64.8% of respondents achieved the CMO's minimum recommended physical activity levels [2]. The odds of meeting CMO's minimum recommended physical activity levels were lower for women than for men and were lower with increasing age. Black or Asian ethnicity was associated with lower odds of participants meeting these levels (see Table 2).

**Table 2:** Associations Between Individual-Level Characteristics and Physical Activity in Adults Residents of Deprived London Neighbourhoods.

Individual Level Variables	Physical activity binary outcome^a^			
Variable/Category	% meeting recommendations	Odds Ratio	95% CI	*P*-value
**Gender**				<0.01
Male	70.70	1.00		
Female	60.20	0.63	0.55, 0.74	
**Age group**				<0.01
16–24	72.30	1.00		
25–34	69.10	0.77	0.62, 0.97	
35–44	65.10	0.66	0.52, 0.83	
45–54	64.40	0.62	0.48, 0.81	
55–64	55.50	0.40	0.30, 0.55	
65+	45.10	0.25	0.19, 0.33	
**Ethnicity**				<0.01
White	65.70	1.00		
Black	63.70	0.75	0.62, 0.90	
Asian	62.90	0.69	0.54, 0.89	
Mixed	69.90	1.14	0.78, 1.66	
Other	66.60	0.91	0.63, 1.31	
**Hope Scale**		1.51	1.37, 1.67	<0.01
**Feels safe on streets alone (Daytime)**				0.03
Very safe	67.00	1.00		
Fairly safe	65.80	0.89	0.73, 1.08	
A bit unsafe	64.50	0.96	0.71, 1.29	
Very unsafe	59.50	0.77	0.54, 1.10	
Never goes out alone	30.80	0.33	0.16, 0.68	
**Feels safe on streets alone (Night-time)**				<0.01
Very safe	67.50	1.00		
Fairly safe	69.10	1.06	0.78, 1.44	
A bit unsafe	68.40	1.09	0.79, 1.42	
Very unsafe	62.80	1.00	0.71, 1.42	
Never goes out alone	48.90	0.68	0.48, 0.97	
**Frequency of meeting with friends**				<0.01
Most days	68.50	1.00		
Once a week or more	67.20	0.93	0.77, 1.11	
Once or twice a month	60.60	0.83	0.65, 1.07	
Less often than once a month	59.00	0.71	0.51, 1.00	
Never	41.50	0.44	0.31, 0.62	
**Frequency of speaking to neighbours**				<0.01
Most days	66.30	1.00		
Once a week or more	66.30	0.79	0.65, 0.96	
Once or twice a month	62.70	0.67	0.51, 0.87	
Less often than once a month	64.00	0.75	0.56, 1.00	
Never	63.00	0.71	0.56, 0.91	

CI = Confidence Interval.

a Adjusted for Age, Gender, Ethnicity.

### Variance in physical activity levels between neighbourhoods

After adjusting for age, gender and ethnicity, the Median Odds Ratio was 1.7 (i.e., if an individual moved to a randomly selected neighbourhood where there was on average a higher probability of meeting the minimum recommended physical activity levels, their median increase in odds of meeting the minimum recommended physical activity levels would be 1.7). Table 3 shows the residual between-neighbourhood variance in physical activity in the empty multilevel model and after adjusting for sociodemographic variables and each neighbourhood-level variable.

**Table 3:** Associations Between Neighbourhood-Level Characteristics and Physical Activity in Adults Residents of Deprived London Neighbourhoods.

	Physical activity binary outcome, adjusted for age, gender and ethnicity						
Variable/Category	Odds Ratio	95% CI	*P*-value	ICC	MOR	Neighbourhood-level variance	PCV
**Model adjusted only for Age, Gender and Ethnicity (Null Model)**				0.08	1.71	0.319	
**Count of large parks within neighbourhood**	3.78	0.52, 27.22	0.19			0.301	5.55%
**Count of greenspaces within neighbourhood**	0.94	0.89, 1.00	0.07			0.312	2.08%
**Pedestrian infrastructure**	0.98	0.93, 1.03	0.47			0.315	1.15%
**Traffic Speed/volume**	0.95	0.91, 1.07	0.69			0.319	0.12%
**Cyclability Index**	1.15	0.67, 1.89	0.66			0.316	0.87%
**Land use mix index**	1.00	1.00, 1.00	0.76			0.318	0.32%
**Greenspace**	1.01	0.98, 1.03	0.46			0.314	1.43%
**Residential**	1.01	0.98, 1.03	0.63			0.317	0.63%
**Commercial**	0.98	0.93, 1.04	0.58			0.316	0.87%
**Transport**	1.00	0.99, 1.02	0.83			0.318	0.20%
**Other land use**	0.98	0.95, 1.02	0.37			0.312	2.28%
**Street connectivity index**	0.11	0.00, 2.74	0.18			0.303	5.10%
**Indices for Multiple Deprivation Crime Score**	1.28	0.97, 1.70	0.08			0.290	9.00%
**Count of incivilities within neighbourhood**	1.23	0.95, 1.57	0.11			0.293	8.23%
**Walking distance to greenspace (from respondent postcodes)**			<0.01			0.347	−8.77%
≤300 metres	1.00						
301–600 meters	0.66	0.50, 0.87					
601–900 meters	0.56	0.39, 0.82					
901–1200 meters	1.01	0.59, 1.72					
**Walking distance to nearest food store (from LSOA centre)**			0.09			0.299	6.35%
≤300 metres	1.00						
301–600 metres	1.30	0.83, 2.05					
601–900 metres	1.28	0.77, 2.14					
>900 metres	1.00	0.31, 3.28					
**Walking time to nearest town centre (from LSOA centre)**			0.52			0.297	6.95%
<3 minutes	1.00						
3–5.9 minutes	1.68	0.64, 4.42					
6–8.9 minutes	1.91	0.81, 4.48					
9–11.9 minutes	1.85	0.70, 4.87					
12–14.9 minutes	1.05	0.33, 3.34					
>15 minutes	2.32	0.73, 7.40					
**Walking distance to nearest sport/leisure facility (from LSOA centre)**			0.09			0.237	25.83%
≤100 metres	1.00						
101–200 metres	1.25	0.78, 1.99					
201–300 metres	0.59	0.32, 1.08					
>300 metres	0.71	0.39, 1.32					

CI = Confidence Interval; ICC = Intraclass Correlation Coefficient; MOR = Median Odds Ratio; PCV = Proportional Change in Variance from Null Model.

### Relationship between individual mental wellbeing, resident-reported neighbourhood characteristics and physical activity

The odds of meeting the CMO's recommended levels of physical activity were higher for respondents reporting higher levels of positive mental wellbeing (Hope Scale, see Table 2). Respondents who reported meeting with friends and speaking to neighbours most regularly had higher odds of achieving the CMO's minimum recommended physical activity levels (Table 2). Perceived measures of the neighbourhood environment (quality of: parks and open spaces; buildings; the environment; neighbourhood peace and quiet; and youth and leisure services and overall neighbourhood satisfaction) were not associated with physical activity. After adjusting for basic sociodemographic characteristics (age, gender and ethnicity), our model showed that respondents who reported feeling safe, both during the day and at night, had higher odds of achieving the recommended physical activity levels (see Table 2).

### Relationship between neighbourhood-audit measures and physical activity

After adjusting for basic sociodemographic factors, the pedestrian infrastructure, traffic speed/volume, cyclability and incivilities indices were not associated with meeting the CMO's minimum recommended physical activity levels (see Table 3). The neighbourhood-audit measures of large parks and greenspaces were also not associated with physical activity levels (see Table 3).

### Relationship between distance to nearest greenspace and physical activity

Respondents whose walking route to the nearest accessible greenspace was 300 m or less had higher odds of achieving recommended physical activity levels compared to respondents with 301–900 m walking routes, but there was no association when compared those living more than 900 m away (see Table 2).

### Relationship between access to sport/leisure facilities, food stores and town centres and physical activity

Walking distances to the nearest available food stores, town centres and sport and leisure were not associated with meeting the minimum recommended physical activity levels (see Table 3).

## Discussion

Our results indicate that individual-level sociodemographic characteristics and self-rated physical health and mental wellbeing are associated with higher odds of achieving the minimum recommended physical activity levels. Positive mental wellbeing was associated with a 1.5 times increase in the odds of meeting recommended levels for each point on the Hope scale. Perceived measures of safety and measures of social capital were also associated with meeting recommended levels. However, the survey items that asked about perceptions of safety contained the option to respond ‘rarely goes out alone’ and while we do not have data on the specific reasons for individuals either choosing not to go out alone or being unable to go out alone, controlling for mobility problems removed the associations.

Respondents who lived within 300 m walking distance of the nearest greenspace suitable for physical activity (within or outside the LSOA in which they lived) had nearly twice the odds of achieving the minimum recommended physical activity levels when compared to those living 600–900 m from the nearest greenspace. In addition to this measure of distance to nearest greenspace, we also examined the presence of large parks or greenspaces within neighbourhoods. These variables were not associated with meeting recommended physical activity levels.

The evidence base for a relationship between greenspace and physical activity is equivocal, with approximately two thirds of previous studies examining associations between proximity to greenspace and physical activity reporting some evidence of a relationship [47]. These studies have used wide-ranging methods to define greenspace accessibility and have seldom focused on residential areas of high deprivation. Our findings suggest that for individuals living in deprived neighbourhoods, the opportunity to directly access useable greenspace a short distance from their home is more important in determining physical activity levels than the presence or proportion of greenspace within their LSOA.

We found evidence of considerable between-neighbourhood variation in the propensity to meet the minimum recommended physical activity levels. However, the measures of neighbourhood-level characteristics used in this study were not associated with the propensity to meet these physical activity levels, and did not explain a substantial amount of the variation in physical activity levels between neighbourhoods.

Limitations of this study include the cross-sectional design which prevents us from making inferences about the direction or existence of a causal link. The use of LSOAs to represent the neighbourhood unit has facilitated the collation of information about neighbourhood characteristics, but the use of census tract areas to define ‘neighbourhoods’ has several limitations [48], [49] and may not correspond to residents' lived neighbourhood [50]. In addition, we have not collected information about workplace physical activity, which may be an important moderator of neighbourhood influences on physical activity.

The physical activity levels reported by respondents to the Well London survey were considerably higher than the national average [3]. While recent studies have suggested that the IPAQ-SF may overestimate physical activity levels when compared to objectives measures such as accelerometers [51], the national average available for comparison is based on self-report recall questions similar to those used in the IPAQ-SF. Furthermore, we have conducted sensitivity analyses using an alternative cut off point for the binary physical activity outcome (7×60 minutes moderate physical activity per week) and also using physical activity MET minutes as a continuous outcome. We found no substantial differences in the relationships presented when examining these alternative outcome measures.

National surveys have shown that low-income groups are less physically active than higher-income groups [3] and multilevel studies have shown that neighbourhood deprivation is associated with lower levels of physical activity [8]. However, our findings suggest high average levels of physical activity in these deprived London neighbourhoods, and that the ease of managing on household income is not associated with physical activity levels (see Table S1.). This suggests that individual-level income and neighbourhood deprivation do not have the expected relationships with physical activity levels in deprived London neighbourhoods. This may be because patterns of deprivation within and between London neighbourhoods are different to patterns of deprivation in the rest of the UK and in the US and Australia, where the majority of previous research has taken place [8].

There is however, substantial variance in physical activity levels between these deprived neighbourhoods. Multilevel social-ecological studies of physical activity have rarely reported information regarding the magnitude of variance in physical activity that can be attributed to variation between neighbourhoods or other geographical areas. The use of the median odds ratio to quantify between-neighbourhood variation in the physical activity outcome provides important information that is seldom reported in multilevel studies of physical activity. This median odds ratio is directly comparable to the odds ratios for individual-level characteristics, suggesting that the median difference in propensity to meet recommended physical activity levels between these 40 neighbourhoods may be similar in magnitude to the differences in propensity between men and women or differences between individuals aged 16–24 and individuals over 55 years old.

The substantial between-neighbourhood variation in physical activity was not explained by the neighbourhood-level variables in this study. This may indicate that neighbourhood-level factors not measured in this study are important determinants of physical activity or that the operationalisation of neighbourhood characteristics in this study was not able to capture the features of the neighbourhoods that are important in determining physical activity levels in this deprived population. The methods used in previous studies to operationalise neighbourhood characteristics have been wide ranging and have seldom focused on deprived neighbourhoods. This may be important in explaining the varying findings in the literature [52], [53]. Future studies may achieve greater specificity by collecting data on crime, incivilities, walkability and cyclability in places where residents prefer to be physically active rather than neighbourhood averages of these variables.

A further potential explanation of our findings is that neighbourhood characteristics influence physical activity through complex and contingent causal pathways that have not been captured in the models presented in this study. Further research is needed to examine such pathways. This research may take the form of analyses that include potential mediating and moderating variables [54] or qualitative research that aims to elucidate causal mechanisms and generate further hypotheses through an understanding of the residents lived experiences [55].

Our observations suggest that in this deprived population, characteristics measured at the individual-level are stronger determinants of physical activity than the neighbourhood-level characteristics examined in this study. However, limited variability in the neighbourhood environments may have restricted the potential to detect neighbourhood-level associations. Variation in the measured neighbourhood characteristics between the deprived neighbourhoods examined in this study and other less deprived neighbourhoods is likely to be greater than the variation between the deprived neighbourhoods alone. Therefore, examination of more heterogeneous neighbourhoods may have yielded different results.

## Supporting Information

Table S1 **Associations Between Individual-Level Characteristics and Physical Activity in Adult Residents of Deprived London Neighbourhoods in 2008.**
(DOC)

Table S2 **Associations Between Neighbourhood-Level Characteristics and Physical Activity in Adult Residents of Deprived London Neighbourhoods in 2008.**
(DOC)

Text S1 **Details of the Specification of the Multiple Imputation Models.**
(DOC)
