# Supplementary material for: Physical Activity in Deprived Communities in London: Examining Individual and Neighbourhood-Level Factors
Source: PLoS One. 2013 Jul 26;8(7):e69472. doi: 10.1371/journal.pone.0069472 (PMC3724838; doi:10.1371/journal.pone.0069472)
Supplement: Table S1 — Associations Between Individual-Level Characteristics and Physical Activity in Adult Residents of Deprived London Neighbourhoods in 2008. (DOC) [file pone.0069472.s001.doc]

Table S1.

| **Individual-level Variables. Adjusted for age, gender and ethnicity (Imputed dataset)** | | | |
| --- | --- | --- | --- |
| **Variable/ Category** | **Odds Ratio** | **95% CI** | ***P*-value** |
| **Employment status** |  |  | <0.01 |
| Employed | 1.00 |  |  |
| Unemployed | 0.74 | 0.60, 0.92 |  |
| Housework/education/retired/ill/other | 0.53 | 0.44, 0.65 |  |
| **Highest education level** |  |  | <0.01 |
| Primary | 1.00 |  |  |
| Secondary (gcse or equivalent) | 1.07 | 0.82, 1.42 |  |
| A' Level of equivalent | 1.17 | 0.87, 1.58 |  |
| Higher (university degree) | 1.55 | 1.14, 2.11 |  |
| Other | 1.13 | 0.51, 2.51 |  |
| **Ease of managing on income** |  |  | 0.27 |
| Very easy | 1.00 |  |  |
| Easy | 0.84 | 0.54, 1.32 |  |
| Neither easy nor difficult | 0.71 | 0.46, 1.11 |  |
| Fairly difficult | 0.66 | 0.42, 1.05 |  |
| Very difficult | 0.70 | 0.45, 1.08 |  |
| **Mobility Problems** |  |  | <0.01 |
| **Problems with usual activities** |  |  | <0.01 |
| **Pain** |  |  | <0.01 |
| **Anxiety** |  |  | <0.01 |
| **Perceived quality of neighbourhood (buildings)** |  |  | 0.10 |
| **Perceived quality of neighbourhood (environment)** |  |  | 0.25 |
| **Perceived quality of neighbourhood (parks and greenspaces)** |  |  | 0.01 |
| **Perceived quality of neighbourhood (youth and leisure services)** |  |  | 0.66 |
| **Neighbourhood Satisfaction** |  |  | 0.05 |
| **Individual Level Variables. Unadjusted (imputed dataset)** | | | |
| **Variable/ Category** | **Odds Ratio** | **95% CI** | ***P*-value** |
| **Gender** |  |  | <0.01 |
| Male | 1.00 |  |  |
| Female | 0.63 | 0.55, 0.71 |  |
| **Age group** |  |  | <0.01 |
| 16-24 | 1.00 |  |  |
| 25-34 | 0.86 | 0.70, 1.06 |  |
| 35-44 | 0.71 | 0.57, 0.89 |  |
| 45-54 | 0.69 | 0.54, 0.89 |  |
| 55-64 | 0.48 | 0.36, 0.63 |  |
| 65+ | 0.31 | 0.24, 0.41 |  |
| **Ethnicity** |  |  | 0.32 |
| White | 1.00 |  |  |
| Black | 0.92 | 0.79, 1.07 |  |
| Asian | 0.88 | 0.73, 1.07 |  |
| Mixed | 1.21 | 0.88, 1.68 |  |
| Other | 1.04 | 0.76, 1.42 |  |
| **Employment status** |  |  | <0.01 |
| Employed | 1.00 |  |  |
| Unemployed | 0.69 | 0.58, 0.83 |  |
| Housework/education/retired/ill/other | 0.49 | 0.42, 0.57 |  |
| **Highest education level** |  |  | <0.01 |
| Primary | 1.00 |  |  |
| Secondary (gcse or equivalent) | 1.51 | 1.20, 1.89 |  |
| A' Level of equivalent | 1.68 | 1.32, 2.13 |  |
| Higher (university degree) | 2.22 | 1.71, 2.88 |  |
| Other | 1.38 | 0.70, 2.72 |  |
| **Ease of managing on income** |  |  | 0.53 |
| Very easy | 1.00 |  |  |
| Easy | 0.83 | 0.55, 1.25 |  |
| Neither easy nor difficult | 0.72 | 0.48, 1.09 |  |
| Fairly difficult | 0.74 | 0.49, 1.12 |  |
| Very difficult | 0.75 | 0.50, 1.12 |  |
| **Hope scale(Odds ratio associated with one point increase on scale)** | 1.38 | 1.27, 1.50 | <0.01 |
| **Mobility Problems** |  |  | <0.01 |
| **Problems with usual activities** |  |  | <0.01 |
| **Pain** |  |  | <0.01 |
| **Anxiety** |  |  | <0.01 |
| **Feels safe on streets alone (Daytime)** |  |  | <0.01 |
| **Feels safe on streets alone (Night-time)** |  |  | <0.01 |
| **Perceived quality of neighbourhood (buildings)** |  |  | <0.01 |
| **Perceived quality of neighbourhood (environment)** |  |  | <0.01 |
| **Perceived quality of neighbourhood (quiet and peaceful )** |  |  | <0.01 |
| **Perceived quality of neighbourhood (parks and open spaces)** |  |  | <0.01 |
| **Perceived quality of neighbourhood (youth and leisure services)** |  |  | 0.26 |
| **Neighbourhood Satisfaction** |  |  | <0.01 |
| **Frequency of meeting with relatives** |  |  | <0.01 |
| **Frequency of meeting with friends** |  |  | <0.01 |
| **Frequency of speaking to neighbours** |  |  | 0.41 |
| **Individual Level Variables. Adjusted for age, gender and ethnicity (Complete cases)** | | | |
| **Variable/ Category** | **Odds Ratio** | **95% CI** | ***P*-value** |
| **Gender** |  |  | <0.01 |
| Male | 1.00 |  |  |
| Female | 0.60 | 0.51, 0.71 |  |
| **Age group** |  |  | <0.01 |
| 16-24 | 1.00 |  |  |
| 25-34 | 0.74 | 0.57, 0.95 |  |
| 35-44 | 0.59 | 0.46, 0.77 |  |
| 45-54 | 0.55 | 0.41, 0.74 |  |
| 55-64 | 0.33 | 0.24, 0.46 |  |
| 65+ | 0.23 | 0.17, 0.31 |  |
| **Ethnicity** |  |  | 0.03 |
| White | 1.00 |  |  |
| Black | 0.80 | 0.65, 0.98 |  |
| Asian | 0.69 | 0.52, 0.92 |  |
| Mixed | 1.20 | 0.79, 1.85 |  |
| Other | 0.86 | 0.57, 1.30 |  |
| **Employment status** |  |  | <0.01 |
| Employed | 1.00 |  |  |
| Unemployed | 0.83 | 0.65, 1.05 |  |
| Housework/education/retired/ill/other | 0.56 | 0.45, 0.69 |  |
| **Highest education level** |  |  | 0.01 |
| Primary | 1.00 |  |  |
| Secondary (gcse or equivalent) | 1.14 | 0.84, 1.56 |  |
| A' Level of equivalent | 1.50 | 1.07,2.10 |  |
| Higher (university degree) | 1.60 | 1.15, 2.24 |  |
| Other | 1.12 | 0.49, 2.54 |  |
| **Ease of managing on income** |  |  | 0.72 |
| Very easy | 1.00 |  |  |
| Easy | 0.83 | 0.49, 1.39 |  |
| Neither easy nor difficult | 0.78 | 0.47, 1.29 |  |
| Fairly difficult | 0.74 | 0.44, 1.23 |  |
| Very difficult | 0.73 | 0.44, 1.22 |  |
| **Hope scale (Odds ratio associated with one point increase on scale)** | 1.43 | 1.27, 1.62 | <0.01 |
| **Mobility Problems** |  |  | <0.01 |
| **Problems with usual activities** |  |  | <0.01 |
| **Pain** |  |  | <0.01 |
| **Anxiety** |  |  | <0.01 |
| **Feels safe on streets alone (Daytime)** |  |  | <0.01 |
| **Feels safe on streets alone (Night-time)** |  |  | <0.01 |
| **Perceived quality of neighbourhood (buildings)** |  |  | 0.38 |
| **Perceived quality of neighbourhood (environment)** |  |  | 0.09 |
| **Perceived quality of neighbourhood (quiet and peaceful )** |  |  | 0.10 |
| **Perceived quality of neighbourhood (parks and open spaces)** |  |  | 0.02 |
| **Perceived quality of neighbourhood (youth and leisure services)** |  |  | 0.39 |
| **Neighbourhood Satisfaction** |  |  | 0.05 |
| **Frequency of meeting with relatives** |  |  | 0.20 |
| **Frequency of meeting with friends** |  |  | <0.01 |
| **Frequency of speaking to neighbours** |  |  | <0.01 |
| **Individual Level Variables. Unadjusted (complete cases)** | | | |
| **Variable/ Category** | **Odds Ratio** | **95% CI** | ***P*-value** |
| **Gender** |  |  | <0.01 |
| Male | 1.00 |  |  |
| Female | 0.60 | 0.52, 0.70 |  |
| **Age group** |  |  | <0.01 |
| 16-24 | 1.00 |  |  |
| 25-34 | 0.81 | 0.64, 1.03 |  |
| 35-44 | 0.63 | 0.49, 0.80 |  |
| 45-54 | 0.63 | 0.47, 0.83 |  |
| 55-64 | 0.41 | 0.30, 0.55 |  |
| 65+ | 0.28 | 0.21, 0.38 |  |
| **Ethnicity** |  |  | 0.24 |
| White | 1.00 |  |  |
| Black | 1.01 | 0.85, 1.19 |  |
| Asian | 0.94 | 0.76, 1.17 |  |
| Mixed | 1.50 | 1.03, 2.19 |  |
| Other | 0.91 | 0.65, 1.29 |  |
| **Employment status** |  |  | <0.01 |
| Employed | 1.00 |  |  |
| Unemployed | 0.75 | 0.61, 0.92 |  |
| Housework/education/retired/ill/other | 0.48 | 0.41, 0.57 |  |
| **Highest education level** |  |  | <0.01 |
| Primary | 1.00 |  |  |
| Secondary (gcse or equivalent) | 1.69 | 1.31, 2.17 |  |
| A' Level of equivalent | 2.40 | 1.84, 3.14 |  |
| Higher (university degree) | 2.40 | 1.85, 3.11 |  |
| Other | 1.03 | 0.51, 2.09 |  |
| **Ease of managing on income** |  |  | 0.45 |
| Very easy | 1.00 |  |  |
| Easy | 0.76 | 0.47, 1.22 |  |
| Neither easy nor difficult | 0.73 | 0.46, 1.15 |  |
| Fairly difficult | 0.75 | 0.47, 1.19 |  |
| Very difficult | 0.67 | 0.42, 1.06 |  |
| **Hope scale (Odds ratio associated with one point increase on scale)** | 1.29 | 1.17, 1.42 | <0.01 |
| **Mobility Problems** |  |  | <0.01 |
| **Problems with usual activities** |  |  | <0.01 |
| **Pain** |  |  | <0.01 |
| **Anxiety** |  |  | <0.01 |
| **Feels safe on streets alone (Daytime)** |  |  | <0.01 |
| **Feels safe on streets alone (Night-time)** |  |  | <0.01 |
| **Perceived quality of neighbourhood (buildings)** |  |  | 0.13 |
| **Perceived quality of neighbourhood (environment)** |  |  | 0.21 |
| **Perceived quality of neighbourhood (quiet and peaceful )** |  |  | 0.08 |
| **Perceived quality of neighbourhood (parks and open spaces)** |  |  | 0.00 |
| **Perceived quality of neighbourhood (youth and leisure services)** |  |  | 0.16 |
| **Neighbourhood Satisfaction** |  |  | 0.24 |
| **Frequency of meeting with relatives** |  |  | 0.00 |
| **Frequency of meeting with friends** |  |  | <0.01 |
| **Frequency of speaking to neighbours** |  |  | 0.30 |

CI = Confidence Interval
