# Supplementary material for: Physical Activity in Deprived Communities in London: Examining Individual and Neighbourhood-Level Factors
Source: PLoS One. 2013 Jul 26;8(7):e69472. doi: 10.1371/journal.pone.0069472 (PMC3724838; doi:10.1371/journal.pone.0069472)
Supplement: Table S2 — Associations Between Neighbourhood-Level Characteristics and Physical Activity in Adult Residents of Deprived London Neighbourhoods in 2008. (DOC) [file pone.0069472.s002.doc]

Table S2.

| **Imputed Dataset** | **Physical activity binary outcome (Unadjusted)** | | | | |
| --- | --- | --- | --- | --- | --- |
| **Variable/ Category** | **Odds Ratio** | **95% CI** | ***P*-value** | **ICC** | **MOR** |
| **Null Model** |  |  |  | 0.08 | 1.66 |
| **Count of large parks within neighbourhood** | 3.02 | 0.48, 19.13 | 0.24 |  |  |
| **Count of greenspaces within neighbourhood** | 0.94 | 0.89, 1.00 | 0.06 |  |  |
| **Pedestrian infrastructure** | 0.98 | 0.92, 1.03 | 0.48 |  |  |
| **Traffic Speed/Volume** | 0.96 | 0.94,1.04 | 0.67 |  |  |
| **Cyclability Index** | 1.15 | 0.71, 1.84 | 0.55 |  |  |
| **Land use mix index** | 1.00 | 1.00, 1.00 | 0.89 |  |  |
| **Greenspace** | 1.01 | 0.99, 1.03 | 0.32 |  |  |
| **Residential** | 1.00 | 0.98, 1.02 | 0.96 |  |  |
| **Commercial** | 0.99 | 0.93, 1.04 | 0.59 |  |  |
| **Transport** | 1.00 | 0.99, 1.02 | 0.90 |  |  |
| **Other land use** | 0.99 | 0.96, 1.02 | 0.43 |  |  |
| **Street connectivity index** | 0.14 | 0.01, 2.86 | 0.20 |  |  |
| **IMD Crime Score** | 1.31 | 1.01, 1.70 | 0.04 |  |  |
| **Count of incivilities within neighbourhood** | 1.22 | 0.96, 1.53 | 0.10 |  |  |
| **Walking distance to nearest food store** |  |  | 0.45 |  |  |
| ≤300 metres | 1.00 |  |  |  |  |
| 301 - 600 metres | 1.21 | 0.80, 1.84 |  |  |  |
| 601 - 900 metres | 1.45 | 0.90, 2.32 |  |  |  |
| > 900 metres | 1.07 | 0.36, 3.21 |  |  |  |
| **Walking time to nearest town centre** |  |  | 0.53 |  |  |
| <3 minutes | 1.00 |  |  |  |  |
| 3 - 5.9 minutes | 1.70 | 0.69, 4.22 |  |  |  |
| 6 - 8.9 minutes | 1.86 | 0.83, 4.13 |  |  |  |
| 9 -11.9 minutes | 1.97 | 0.80, 4.89 |  |  |  |
| 12 - 14.9 minutes | 1.16 | 0.39, 3.43 |  |  |  |
| >15 minutes | 2.31 | 0.78, 6.86 |  |  |  |
| **Walking distance to nearest sport/leisure facility** |  |  | 0.03 |  |  |
| ≤100 metres | 1.00 |  |  |  |  |
| 101 - 200 metres | 1.25 | 0.82, 1.91 |  |  |  |
| 201 - 300 metres | 0.57 | 0.33, 1.00 |  |  |  |
| >300 metres | 0.70 | 0.40, 1.23 |  |  |  |
| **Walking distance to nearest greenspace** |  |  | <0.01 |  |  |
| ≤300 metres | 1.00 |  |  |  |  |
| 301 - 600 metres | 0.73 | 0.61, 0.87 | <0.01 |  |  |
| 601-900 metres | 0.71 | 0.59, 0.86 | <0.01 |  |  |
| 900 - 1200 metres | 1.35 | 1.01, 1.80 | 0.04 |  |  |
| **Complete Cases** | **Physical activity binary outcome (Unadjusted)** | | | | |
| **Variable/ Category** | **Odds Ratio** | **95% CI** | ***P*-value** | **ICC** | **MOR** |
| **Null Model** |  |  |  | 0.08 | 1.64 |
| **Count of large parks within neighbourhood** | 3.28 | 0.51, 21.03 | 0.21 |  |  |
| **Count of greenspaces within neighbourhood** | 0.92 | 0.87, 0.98 | 0.00 |  |  |
| **Pedestrian infrastructure** | 0.98 | 0.93, 1.05 | 0.61 |  |  |
| **Traffic Speed/Volume** | 0.97 | 0.94, 1.04 | 0.47 |  |  |
| **Cyclability Index** | 1.13 | 0.74, 1.33 | 0.60 |  |  |
| **Land use mix index** | 1.00 | 1.00, 1.00 | 0.97 |  |  |
| **Greenspace** | 1.00 | 0.98, 1.03 | 0.71 |  |  |
| **Residential** | 1.01 | 0.98, 1.03 | 0.57 |  |  |
| **Commercial** | 0.98 | 0.93, 1.04 | 0.50 |  |  |
| **Transport** | 1.00 | 0.99, 1.02 | 0.64 |  |  |
| **Other land use** | 0.97 | 0.94, 1.00 | 0.08 |  |  |
| **Street connectivity index** | 0.49 | 0.02, 10.50 | 0.64 |  |  |
| **IMD Crime Score** | 1.33 | 1.02, 1.73 | 0.03 |  |  |
| **Count of incivilities within neighbourhood** | 1.22 | 0.96, 1.55 | 0.09 |  |  |
| **Walking distance to nearest food store** |  |  | 0.36 |  |  |
| ≤300 metres | 1.00 |  |  |  |  |
| 301 - 600 metres | 1.40 | 0.92, 2.13 |  |  |  |
| 601 - 900 metres | 1.33 | 0.83, 2.13 |  |  |  |
| > 900 metres | 0.93 | 0.31. 2.80 |  |  |  |
| **Walking time to nearest town centre** |  |  | 0.25 |  |  |
| <3 minutes | 1.00 |  |  |  |  |
| 3 - 5.9 minutes | 2.45 | 1.06, 5.67 |  |  |  |
| 6 - 8.9 minutes | 2.31 | 1.11, 4.84 |  |  |  |
| 9 -11.9 minutes | 2.23 | 0.96, 5.16 |  |  |  |
| 12 - 14.9 minutes | 1.57 | 0.57, 4.30 |  |  |  |
| >15 minutes | 2.89 | 1.05, 7.98 |  |  |  |
| **Walking distance to nearest sport/leisure facility** |  |  | 0.43 |  |  |
| ≤100 metres | 1.00 |  |  |  |  |
| 101 - 200 metres | 1.16 | 0.70 1.92 |  |  |  |
| 201 - 300 metres | 0.67 | 0.35, 1.28 |  |  |  |
| >300 metres | 0.86 | 0.44, 1.69 |  |  |  |
| **Walking distance to nearest greenspace** |  |  | <0.01 |  |  |
| ≤300 metres | 1.00 |  |  |  |  |
| 301 - 600 metres | 0.70 | 0.58, 0.85 |  |  |  |
| 601-900 metres | 0.88 | 0.72, 1.09 |  |  |  |
| 900 - 1200 metres | 1.27 | 0.93, 1.73 |  |  |  |
| **Complete Cases** | **Physical activity binary outcome (Adjusted for age, gender and ethnicity)** | | | | |
| **Variable/ Category** | **Odds Ratio** | **95% CI** | ***P*-value** | **ICC** | **MOR** |
| **Model adjusted only for Age, Gender and Ethnicity** |  |  |  | 0.09 | 1.71 |
| **Count of large parks within neighbourhood** | 3.70 | 0.47, 28.88 | 0.21 |  |  |
| **Count of greenspaces within neighbourhood** | 0.94 | 0.88, 1.01 | 0.11 |  |  |
| **Pedestrian infrastructure** | 0.98 | 0.94, 1.06 | 0.62 |  |  |
| **Traffic Speed/Volume** | 0.96 | 0.93, 1.04 | 0.48 |  |  |
| **Cyclability Index** | 1.33 | 0.73, 1.42 | 0.64 |  |  |
| **Land use mix index** | 1.00 | 1.00. 1.00 | 0.83 |  |  |
| **Greenspace** | 1.00 | 0.98, 1.03 | 0.80 |  |  |
| **Residential** | 1.01 | 0.99, 1.04 | 0.33 |  |  |
| **Commercial** | 0.98 | 0.92, 1.04 | 0.51 |  |  |
| **Transport** | 1.01 | 0.99, 1.02 | 0.53 |  |  |
| **Other land use** | 0.97 | 0.94, 1.00 | 0.07 |  |  |
| **Street connectivity index** | 0.09 | 0.00, 2.44 | 0.15 |  |  |
| **IMD Crime Score** | 1.24 | 0.92, 1.67 | 0.16 |  |  |
| **Count of incivilities within neighbourhood** | 1.24 | 0.96, 1.61 | 0.10 |  |  |
| **Walking distance to nearest food store** |  |  | 0.32 |  |  |
| ≤300 metres | 1.00 |  |  |  |  |
| 301 - 600 metres | 1.54 | 0.97, 2.44 |  |  |  |
| 601 - 900 metres | 1.18 | 0.70, 1.98 |  |  |  |
| > 900 metres | 0.91 | 0.27, 3.03 |  |  |  |
| **Walking time to nearest town centre** |  |  | 0.24 |  |  |
| <3 minutes | 1.00 |  |  |  |  |
| 3 - 5.9 minutes | 2.55 | 1.03, 6.35 |  |  |  |
| 6 - 8.9 minutes | 2.51 | 1.13, 5.59 |  |  |  |
| 9 -11.9 minutes | 2.13 | 0.86, 5.28 |  |  |  |
| 12 - 14.9 minutes | 1.57 | 0.53, 4.69 |  |  |  |
| >15 minutes | 3.10 | 1.04, 9.26 |  |  |  |
| **Walking distance to nearest sport/leisure facility** |  |  | 0.46 |  |  |
| ≤100 metres | 1.00 |  |  |  |  |
| 101 - 200 metres | 1.21 | 0.70, 2.09 |  |  |  |
| 201 - 300 metres | 0.70 | 0.35, 1.41 |  |  |  |
| >300 metres | 0.79 | 0.38, 1.64 |  |  |  |
| **Walking distance to nearest greenspace** |  |  | <0.01 |  |  |
| ≤300 metres | 1.00 |  |  |  |  |
| 301 - 600 metres | 0.62 | 0.46, 0.83 |  |  |  |
| 601-900 metres | 0.60 | 0.40, 0.90 |  |  |  |
| 900 - 1200 metres | 0.94 | 0.52, 1.70 |  |  |  |

CI = Confidence Interval; ICC= Intraclass Correlation Coefficient; MOR = Median Odds Ratio
