# Supplementary material for: Physical Activity in Deprived Communities in London: Examining Individual and Neighbourhood-Level Factors
Source: PLoS One. 2013 Jul 26;8(7):e69472. doi: 10.1371/journal.pone.0069472 (PMC3724838; doi:10.1371/journal.pone.0069472)
Supplement: Text S1 — Details of the Specification of the Multiple Imputation Models. (DOC) [file pone.0069472.s003.doc]

Text S1.

| For the physical activity outcome measure imputation prediction models were specified to predict a value for those that are missing from the household survey dataset. The Well London survey dataset contains a large number of variables and only those variables which *a priori* are thought to plausibly hold substantial information about the outcomes or key covariates were used in the imputation model. For the physical activity outcome each IPAQ-SF item was imputed separately and the overall composite outcome score calculated from these imputed items. |
| --- |
| An indicator for LSOA was included in the imputation to account for the clustering in the data. There are no established computer algorithms for multilevel/random effects imputation models for binary categorical outcomes, therefore a fixed effects multiple imputation model in the current version of *ice* for Stata was used. |
| The variables included in the imputations models for each IPAQ-SF composite item (n=7) were: The other 6 IPAQ score composite items; age; gender; ethnicity; level of education achieved; marital status; housing tenure; ease of managing on household income; smoking; alcohol consumption; reports cardiovascular disease diagnosis; reports diabetes diagnosis; reports respiratory condition diagnosis; reports mobility problems; weight; body mass index; waist circumference; visit to GP in last 12 months. |
